# Random forest-based modelling to detect biomarkers for prostate cancer progression

**DOI:** 10.1186/s13148-019-0736-8

**Published:** 2019-10-22

**Authors:** Reka Toth, Heiko Schiffmann, Claudia Hube-Magg, Franziska Büscheck, Doris Höflmayer, Sören Weidemann, Patrick Lebok, Christoph Fraune, Sarah Minner, Thorsten Schlomm, Guido Sauter, Christoph Plass, Yassen Assenov, Ronald Simon, Jan Meiners, Clarissa Gerhäuser

**Affiliations:** 1 Cancer Epigenomics, German Cancer Research Center (DKFZ), 69120 Heidelberg, Germany; 2 Department of Pathology, University Medical Center Hamburg-Eppendorf, 20246 Hamburg, Germany; 3 Department of Urology, Charité Universitätsmedizin Berlin, 10117 Berlin, Germany; 4 German Cancer Consortium (DKTK), 69120 Heidelberg, Germany; 5 General, Visceral and Thoracic Surgery Department and Clinic, University Medical Center Hamburg-Eppendorf, 20246 Hamburg, Germany

## Abstract

**Background:**

The clinical course of prostate cancer (PCa) is highly variable, demanding an individualized approach to therapy. Overtreatment of indolent PCa cases, which likely do not progress to aggressive stages, may be associated with severe side effects and considerable costs. These could be avoided by utilizing robust prognostic markers to guide treatment decisions.

**Results:**

We present a random forest-based classification model to predict aggressive behaviour of prostate cancer. DNA methylation changes between PCa cases with good or poor prognosis (discovery cohort with *n* = 70) were used as input. DNA was extracted from formalin-fixed tumour tissue, and genome-wide DNA methylation differences between both groups were assessed using Illumina HumanMethylation450 arrays. For the random forest-based modelling, the discovery cohort was randomly split into a training (80%) and a test set (20%). Our methylation-based classifier demonstrated excellent performance in discriminating prognosis subgroups in the test set (Kaplan-Meier survival analyses with log-rank *p* value < 0.0001). The area under the receiver operating characteristic curve (AUC) for the sensitivity analysis was 95%. Using the ICGC cohort of early- and late-onset prostate cancer (*n* = 222) and the TCGA PRAD cohort (*n* = 477) for external validation, AUCs for sensitivity analyses were 77.1% and 68.7%, respectively. Cancer progression-related DNA hypomethylation was frequently located in ‘partially methylated domains’ (PMDs)—large-scale genomic areas with progressive loss of DNA methylation linked to mitotic cell division. We selected several candidate genes with differential methylation in gene promoter regions for additional validation at the protein expression level by immunohistochemistry in > 12,000 tissue micro-arrayed PCa cases. Loss of ZIC2 protein expression was associated with poor prognosis and correlated with significantly shorter time to biochemical recurrence. The prognostic value of ZIC2 proved to be independent from established clinicopathological variables including Gleason grade, tumour stage, nodal stage and prostate-specific-antigen.

**Conclusions:**

Our results highlight the prognostic relevance of methylation loss in PMD regions, as well as of several candidate genes not previously associated with PCa progression. Our robust and externally validated PCa classification model either directly or via protein expression analyses of the identified top-ranked candidate genes will support the clinical management of prostate cancer.

## Background

Prostate cancer (PCa) is the second most prevailing cancer in the male population worldwide, an estimated 1.28 Mio. newly diagnosed cases and 350,000 cancer-related deaths in 2018 [1]. Although the aetiology of prostate cancer is controversial, it is likely to result from accumulating DNA damage in stress-exposed ageing prostate epithelial cells [2]. Specifically, chromosomal rearrangements and oncogene fusion genes in these cells are driven by androgens [3, 4]. Despite a large number of studies that have suggested a multitude of candidate prognostic markers in prostate cancer, none of these genes has proven to be superior over the established histological prognostic factors including tumour stage and Gleason grade. Localized prostate cancer with low Gleason score usually remains indolent, requiring only active surveillance or minimal treatment. Nevertheless, many patients may be over-treated with associated side effects and substantial costs [5]. There is, therefore, general agreement that novel specific biomarkers for the diagnosis and prognosis of prostate cancer are needed for an efficient clinical management of this disease [6, 7].

Recent high-resolution genome-wide studies have significantly improved our understanding of chromosomal and genetic alterations associated with prostate cancer development, such as the androgen-driven formation of gene fusions between the transmembrane serine protease TMPRSS2 and a member of the oncogenic ETS transcription factor family like ERG in about 50% of all prostate cancer cases, and frequent loss of the tumour suppressor gene PTEN [4, 8, 9]. These events affect signalling pathways and lead to alterations in gene expression programs that have been used for the development of gene signatures (genomic classifiers) as biomarkers for the prediction of prostate cancer prognosis [10]. Although several studies have demonstrated some prognostic value of gene expression-based signatures, due to the limited stability of RNA and often low quality when extracted from formalin-fixed paraffin-embedded (FFPE) material, protein- or DNA-based methods might be superior to RNA expression profiles for biomarker development.

There is substantial evidence that genetic defects in prostate cancer are complemented or even preceded by epigenetic aberrations such as DNA methylation [11]. Novel technologies based on genome-wide screens for aberrant DNA methylation and epigenetic gene silencing, including the widely used Illumina 450k Beadchip arrays, have allowed identification of hundreds of genes aberrantly methylated during prostate cancer development [3, 8, 11, 12]. These cancer-specific epigenetic alterations have been shown to enable the development of methylation-based assays to distinguish between benign and malignant tissue and to predict the course of the disease [11, 13, 14].

In recent years, machine-learning techniques became widely used in modern molecular research to build predictive models [15]. Random forest [16] is an ensemble learning method based on the construction of many classification trees. Main benefits of the method are its robustness against overfitting, user-friendliness and the easy interpretation of the model [16].

Our goal was to use random forest-based modelling of DNA methylation alterations to develop a classifier predicting the outcome of prostate cancer. In addition, the tight connection of DNA methylation events with gene expression allowed us to utilize immunohistochemistry (IHC), a universally available tool in diagnostic laboratories, on tissue microarrays of thousands of clinically well-annotated samples to validate ZIC2 as a prognostic protein biomarker independent of established clinicopathological variables.

## Results

### Differential methylation analysis

To identify methylation alterations associated with PCa aggressiveness, we used a discovery cohort of 70 PCa cases (Table 1) with good (organ-confined disease and lack of recurrence for at least 5 years) or poor prognosis (systemic presence of metastatic disease, indicated by biochemical PSA-based recurrence within 3 years and no response to local radiation therapy) for genome-wide methylation analyses using Illumina 450k arrays. The two groups showed differences in preoperative PSA levels (*p* = 1.2 × 10^− 6^) and survival rates. Patients in the poor prognosis group suffered from rapid BCR, with a median disease-free survival of 3.8 months.

**Table 1:** Clinical characteristics of the discovery cohort

	Good prognosis^a^	Poor prognosis^a^
*n*	35	35
Age (mean ± sd)	62.7 ± 5.6	65 ± 6.6
Pretreatment PSA (ng/ml)	6.86 ± 3.4	28.3 ± 22.3
Stage (path. T)		
pT2	35	0
pT3a	0	3
pT3b	0	31
pT4	0	1
Gleason score		
3 + 3	15	0
3 + 4	17	6
4 + 3	3	15
4 + 4	0	4
4 + 5	0	7
5 + 4	0	3

^a^Good prognosis defined as an organ-confined disease (pT2) and lack of biochemical (PSA-based) recurrence (BCR) for at least 5 years. Poor prognosis defined as systemic presence of metastatic disease, indicated by recurrence within 3 years and no response to local radiation therapy

**Fig. 1:**
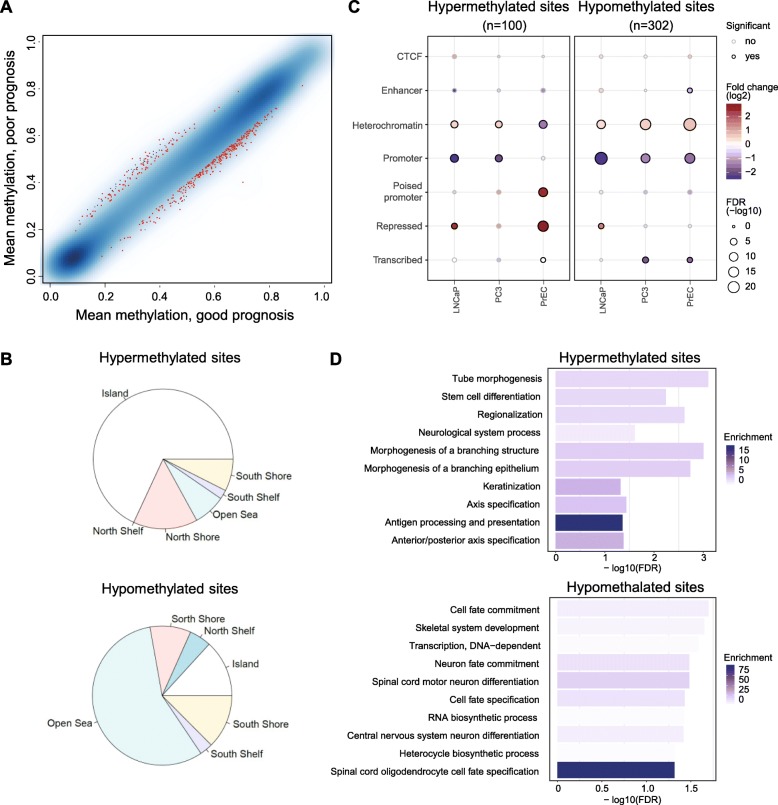
Differential methylation analysis. **a** Mean methylation values of the good and poor prognosis groups in a smoothed colour density representation plot. Sites with FDR-corrected *p* values < 0.2 and absolute beta value difference > 0.1 are marked in red. **b** Distribution of the localization of differentially methylated CpG sites (DMS) hypermethylated (*n* = 100, top) or hypomethylated (*n* = 302, bottom) in the poor prognosis group relative to the good prognosis group, in relation to CpG islands. **c** Enrichment analysis of the hypermethylated (left) and hypomethylated (right) DMS using 7-state ChromHMM data for PC3 and LnCaP tumour cell lines and prostate epithelial cells (PrEC) [9]. The size of the circles demonstrates the significance of enrichment, while their colour represents the strength and the direction of the enrichment (red: enriched, blue: depleted). The black circle outline indicates significant results. **d** Pathway analysis of genes associated with the hypermethylated (top) and hypomethylated (bottom) DMS. GREAT tool was used to assign genes to CpG sites. The shade of blue shows the significance of the enrichment, while the bars represent the strength

**Fig. 2:**
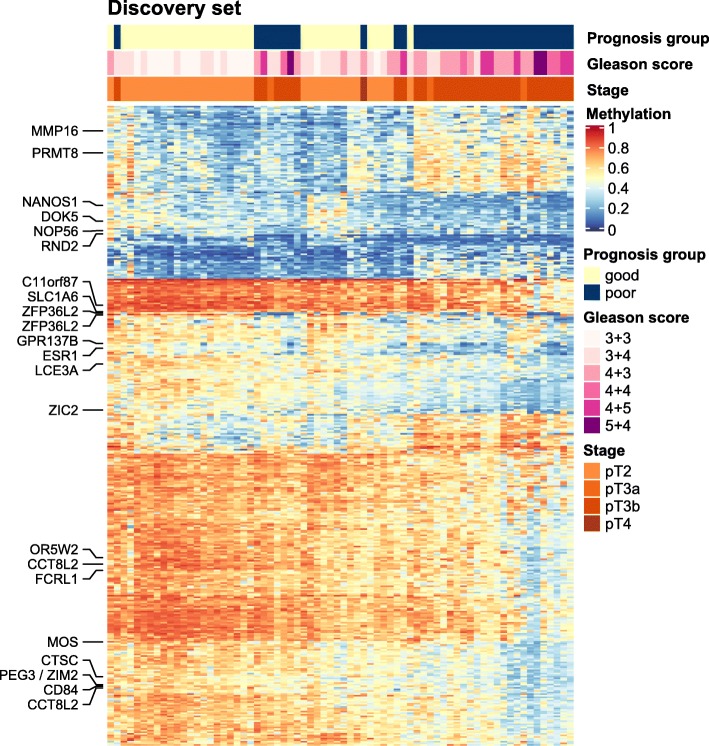
Methylation heatmap of the selected CpG sites in the discovery cohort. Each column represents a sample with predicted good or poor prognosis, while rows represent selected differentially methylated CpG sites. Annotations on the left side indicate top-ranked candidate genes associated with most informative CpG sites. Low and high methylation beta values in a range from 0 to 1 are shown in a blue to red colour scale. Hierarchical clustering was done on Euclidian distance between samples and sites. BCR: PSA-based biochemical recurrence

After adjusting for age at diagnosis and tumour purity (based on the samples’ basal, stromal and immune cell contents computed from DNA methylation data using the PEPCI R-package [9] (Additional file 1: Table S1)), we selected 402 differentially methylated CpG sites (DMS, with minimum 10% absolute methylation difference, FDR-adjusted *p* value < 0.2) (Fig. 1a). Of these, 302 DMS lost methylation in the poor prognosis group compared to the good prognosis group, and 100 DMS gained methylation (Fig. 2). Hypermethylated DMS were mainly localized in CpG islands, shores and shelves, while DMS with loss in methylation were mostly located in intergenic (open sea) regions (Fig. 1b). To characterize the enrichment of DMS in specific genomic regions, we used the EpiAnnotator tool and chromatin state information (ChromHMM data) for normal prostate (PrEC) and prostate cancer (PC3, LnCAP) cell lines [9]. DMS with hypermethylation in aggressive PCa were enriched in poised promoters and repressed regions in normal PrCE cells. In both prostate cancer cell lines, these regions were marked as heterochromatin, indicating remodelling of the 3D chromatin structure during carcinogenesis. DMS that lost methylation in aggressive tumours showed enrichment for heterochromatic, often gene-poor regions in normal prostate as well as in prostate cancer cell lines (Fig. 1c).

We observed only minor differences in enrichment between the androgen-responsive cell line LNCaP and AR-independent PC3 cells. Still, we explored the proximity of the genes associated with DMS to androgen receptor binding sites (ARBS), using a list of consensus ARBS (*n* = 8162) derived from Stelloo et al. [17]. For > 90% of the genes, the most proximal ARBS was located > 10 kb away from the transcription start sites (TSSs) (Additional file 2: Table S2), and none of the DMS directly overlapped with an ARBS. These findings indicated that androgen signalling was not the major driver underlying differential methylation between the two prognosis groups.

Poised promoters that significantly overlapped with hypermethylated DMS in PrEC synchronously bear activating and repressive histone marks at the transcription start site and are often associated with cell fate determination and differentiation [18]. In line with these observations, a GREAT-based pathway analysis [19] of genes associated with hypermethylated DMS showed enrichment of developmental processes (Fig. 1d).

### Random forest model

We applied random forest-based modelling to rank the selected DMS according to their discriminative power (for details, see description in the “Methods” section). In addition to the DMS, our recently developed Purity-Adjusted Epigenetic Prostate Cancer Index (PEPCI) of tumour aggressiveness [9] was included in the model. Mean PEPCI was significantly different between the two prognosis groups of the discovery cohort (*t* test *p* value = 0.03). Using a cut-off of 69.1 to define PEPCI-low and PEPCI-high tumours (as described in [9]), the aggressivity score stratified the discovery cohort according to PSA recurrence-free survival (log-rank *p* value = 0.045) (Additional file 3: Figure S1).

For the random forest-based modelling, the discovery cohort was randomly split into a training (80% randomly selected samples) and a test set (20% randomly selected samples). The model was trained on the training set, with 10,000 trees. Prediction accuracy was then measured on the test set. For variable selection, DMS were ranked based on mean decrease in accuracy and Gini scores [20] (complete list of CpG sites in the model, as well as importance scores in Additional file 2: Table S2). The Gini score indicates how often a random sample from the test set would be incorrectly categorized as having good or poor prognosis if the samples were randomly distributed [20].

**Fig. 3:**
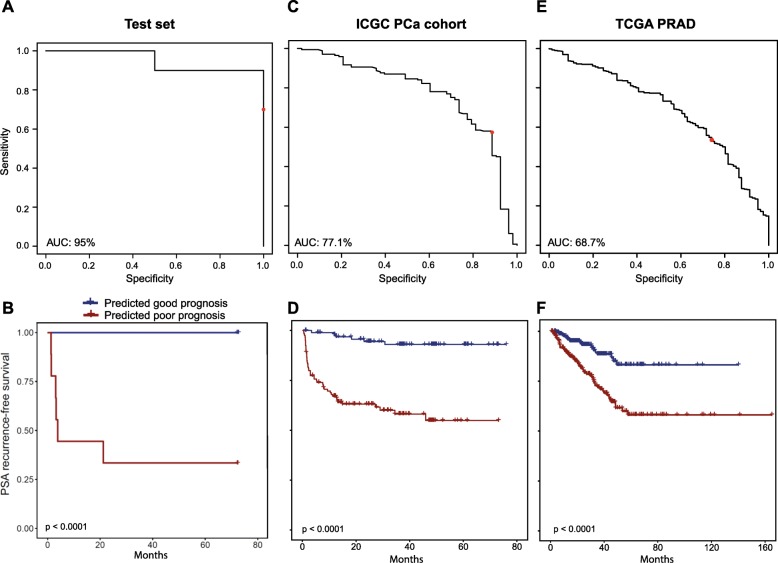
Performance analysis of the model in the test and validation datasets. **a**, **c**, **e** ROC curve analysis of the model’s performance in the test set (**a**), ICGC PCa cohort (**c**) and TCGA PRAD cohort (**e**). The *x*-axis shows the model’s specificity while the *y*-axis shows the sensitivity. Red dot represents the performance of the model when a cut-off of 0.5 is used during classification. **b**, **d**, **f** Kaplan-Meier curves using PSA recurrence-free survival as an outcome in the test set (**b**), in the ICGC PCa cohort (**d**) and in the TCGA-PRAD cohort (**f**), based on the predicted prognostic categories (blue: good prognosis, red: poor prognosis). *p* values were calculated using log-rank test

The random forest model showed an error of 14.81% on the training set (*n* = 56), with better prediction for the poor prognosis subgroup (Additional file 4: Figure S2). On the test set (*n* = 14), the model showed an error rate of 18.8%, with an area under the receiver operating characteristic (ROC) curve (AUC) of 95% (Fig. 3a). A Kaplan-Meier plot indicated excellent stratification of the subgroups of the test set predicted to have good or poor prognosis (log-rank *p* value < 0.0001, Fig. 3b). We applied our model to two independent PCa cohorts for validation of the good prediction rate. We were able to validate our results using the ICGC PCa cohort of early- and late-onset prostate cancer (*n* = 222) [9]. The AUC for the sensitivity analysis was 77.1% (Fig. 3c). With an AUC of 99.7%, the model demonstrated excellent performance when we only used a subset of the cohort based on the same selection criteria as in our discovery cohort (*n* = 63). With the TCGA PRAD cohort (*n* = 477, Table 2), we observed an AUC of 68.7% (Fig. 3e), while the AUC was 77.5% with a preselected subset (*n* = 84, Table 2**)**. Our classifier efficiently stratified both validation cohorts according to PSA recurrence-free survival (log-rank *p* value < 0.0001 for both cohorts) (Fig. 3d, f and Additional file 5: Figure S3). In the ICGC dataset, our model proved to be an independent predictor of recurrence-free survival, when the Gleason score was included in the model (Cox regression *p* = 0.011).

**Table 2:** Clinical characteristics of the TCGA PRAD cohort and the preselected subcohort

	TCGA PRAD	TCGA PRAD subcohort	
	Full cohort	Good prognosis^a^	Poor prognosis^a^
*n*	477	27	57
Age (mean ± sd)	61.06 ± 6.9	57.23 ± 6.7	62.46 ± 6
Stage (path. T)			
n.a.	6	0	0
pT2a	13	1	0
pT2b	6	3	0
pT2c	161	23	0
pT3a	153	0	24
pT3b	129	0	31
pT4	9	0	2
Gleason score			
6	43	6	0
7	243	17	12
8	61	2	8
9	127	2	36
10	3	0	1

*n.a.* not available

^a^Good prognosis defined as lack of BCR for at least 5 years and stage < pT3. Poor prognosis defined as BCR within 3 years and stage ≥ pT3

### Candidate selection

Based on their localization in regulatory regions and distance from TSS, DMS were associated with genes [19]. The genes were ranked to select the top 20 candidates for confirmatory analyses based on immunohistochemistry (IHC), as further described below (Table 3, with individual Kaplan-Meier curves for the candidate gene-related CpG sites and the full model in Additional file 3: Figure S1).

**Table 3:** Top 20 ranked candidate CpG probes and associated genes

CG probe names	Chr.	Location	Mean decrease in accuracy	Mean decrease in Gini	ChromHMM PC3	ChromHMM PrEC	ChromHMM LNCaP	Gene symbol	Distance from TSS (bp)	Mean meth. good progn.	Mean meth. poor progn.
cg07443748	chr22	17073594	0.002194	0.221948	Heterochromatin	Heterochromatin	Heterochromatin	CCT8L2	105	0.585	0.434
cg18146506	chr20	2633340	0.002382	0.193534	Promoter	Promoter	Promoter	NOP56	550	0.430	0.327
cg05766896	chr1	157789933	0.001993	0.192736	Heterochromatin	Heterochromatin	Heterochromatin	FCRL1	− 39	0.657	0.508
cg16734913	chr11	55681277	0.001621	0.167347	Heterochromatin	Heterochromatin	Heterochromatin	OR5W2	780	0.688	0.567
cg16876647	chr2	43451842	0.002281	0.15899	Promoter	Promoter	Heterochromatin	ZFP36L2	1905	0.703	0.401
cg24100636	chr12	3600087	0.001015	0.145156	Heterochromatin	Promoter	Heterochromatin	PRMT8	− 314	0.280	0.446
cg16377872	chr19	15084823	0.00141	0.143848	Heterochromatin	Heterochromatin	Heterochromatin	SLC1A6	− 1094	0.747	0.641
cg27212232	chr20	53091600	0.001276	0.143601	Heterochromatin	Heterochromatin	Heterochromatin	DOK5	− 535	0.407	0.301
cg02601618	chr22	17075046	0.002015	0.134821	Heterochromatin	Heterochromatin	Heterochromatin	CCT8L2	− 1347	0.696	0.565
cg12092201	chr2	43451775	0.001586	0.122542	Promoter	Promoter	Heterochromatin	ZFP36L2	1972	0.473	0.285
cg12818557	chr8	89340139	0.00084	0.122408	Heterochromatin	Promoter	Promoter	MMP16	− 423	0.329	0.503
cg04211581	chr6	152011656	0.000859	0.117083	CTCF	Heterochromatin	Transcribed	ESR1	26	0.458	0.317
cg24690071	chr13	100635352	0.001171	0.116259	Poised promoter	Repressed	Promoter	ZIC2	1327	0.483	0.322
cg00874055	chr1	236306673	0.001063	0.115871	Promoter	Promoter	Poised promoter	GPR137B	842	0.487	0.342
cg10562114	chr10	120790501	0.000745	0.112334	Promoter	Promoter	Promoter	NANOS1	1274	0.353	0.230
cg26135325	chr1	152595322	0.000427	0.104978	Heterochromatin	Heterochromatin	Heterochromatin	LCE3A	256	0.540	0.431
cg00343414	chr11	109292465	0.000626	0.10478	Heterochromatin	Heterochromatin	Heterochromatin	C11orf87	− 380	0.795	0.691
cg25458871	chr19	57352584	0.000731	0.104688	Heterochromatin	Heterochromatin	Heterochromatin	PEG3	− 521	0.532	0.428
cg25458871	chr19	57352584	0.000731	0.104688	Heterochromatin	Heterochromatin	Heterochromatin	ZIM2	− 488	0.532	0.428
cg16118839	chr11	88069169	0.000997	0.103679	Promoter	Enhancer	Heterochromatin	CTSC	1785	0.526	0.408
cg16776350	chr1	160549158	0.000666	0.102419	Heterochromatin	Heterochromatin	Heterochromatin	CD84	135	0.590	0.459
cg00636390	chr8	57027352	0.000914	0.101489	Heterochromatin	Repressed	Repressed	MOS	− 812	0.616	0.485
cg17225407	chr17	41176954	0.000767	0.101076	Poised promoter	Poised promoter	Heterochromatin	RND2	− 303	0.388	0.287

Comparison with recently published whole genome bisulfite sequencing data (WGBS) for prostate cancer [21] revealed that about 60% of the top DMS (associated with *C11orf87*, *CCT8L2*, *CD84*, *CTSC*, *DOK5*, *FCRL1*, *LCE3A*, *MMP16*, *MOS*, *OR5W2*, *PEG3/ZIM2* and *SLC1A6*) were located in so-called partially methylated domains (PMDs) (Additional file 6: Figure S4). PMDs are genomic regions of several hundred kilobases to few megabases in length that are associated with heterochromatic areas in the nuclear periphery, replicated late during cell cycle progression and progressively losing methylation. In tumours, stronger hypomethylation in PMDs was significantly associated with higher genome-wide somatic mutation densities [22], supporting our findings of commonly more loss of methylation in more aggressive PCa compared to the good prognosis group.

Beside the PMD-associated DMS, we also identified DMS with focal changes in methylation in gene promoter regions. The gene of matrix metalloproteinase 16 (MMP16), a proteolytic enzyme involved in the development of PCa progression and metastases [23], is located in a frequent PMD. However, we identified cg12818557 located in the promoter region of *MMP16* as a strong predictor, with almost 20% methylation gain in the poor compared to the good prognosis group.

Gain of methylation at cg00874055 in the promoter region of *GPR137B* (G protein-coupled receptor 137B) was inversely correlated (rho = − 0.497, *p* = 1.85 × 10^−6^) with mRNA expression (data from ICGC EOPC cohort). GPR137B upregulation is linked to aggressive forms of pancreatic cancer [24] and associated with increased proliferation in various cancer types but has not been identified as a PCa biomarker yet.

Similarly, nucleolar protein *NOP56* (cg18146506) and protein arginine methyltransferase *PRMT8* (cg24100636) with hypomethylated promoter DMS were were identified as biomarkers for multiple cancer types [25, 26]. Arginine methylation is relevant for various cellular processes, including DNA repair, RNA transcription, signal transduction, protein compartmentalization, and possibly protein translation [27]. We also identified a DMS (cg17225407) in the promoter of *RND2*, a relatively unexplored member of the Rho GTPase family [28], with loss in methylation in the poor prognosis group compared to the good prognosis group. Cg04211581 is located only 26 bps from the TSS of *ESR1*. *ESR1* encodes oestrogen receptor alpha (ERα), the role of which has been proposed in PCa; however, it is still controversial [29]. Interestingly, we identified an ARBS in close vicinity (distance < 1 kbp) of the DMS.

Three DMS affected the promoter region of zinc finger proteins. Two sites were located in the promoter of *ZFP36L2* (cg16876647, cg12092201), while one CpG site (cg24690071) was located in a poised promoter of *ZIC2*. *ZFP36L2* encodes a CCH-type zinc finger protein, which is regulated by the cell-cycle, might play a role in DNA damage response [30] and inhibit cell proliferation [31]. In PCa, ZFP36L2 upregulation was associated with the transcription factor Runx2 and poor prognosis [32]. ZIC2 belongs to a family of transcription factors involved in neuroectodermal development. Elevated ZIC2 mRNA expression was described in high Gleason prostate cancer [33].

Our results highlight the prognostic relevance of methylation loss in PMD regions, as well as of several candidate genes not previously associated with PCa. The influence of the methylation changes of these candidates DMS on gene or protein expression and the impact on prostate carcinogenesis needs to be experimentally confirmed in mechanistic chromatin conformation and gain- and loss-of-function studies.

### Candidate validation

**Fig. 4:**
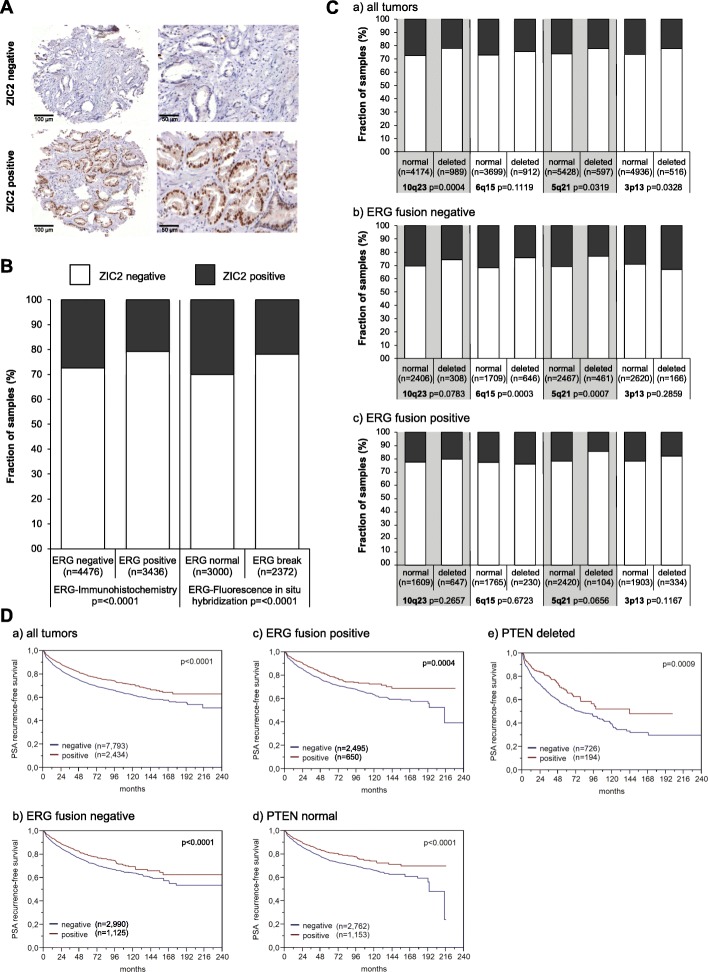
ZIC2 immunostaining in > 12,000 micro-arrayed PCa cases. **a** Examples of negative (no nuclear staining, upper panels) and strong staining (lower panels). **b** Association between ZIC2 immunostaining results and the ERG-status determined by IHC and FISH analysis. **c** Association between ZIC2 immunostaining and deletions of 10q23 (PTEN), 6q15 (MAP 3 K7), 5q21 (CHD1) and 3p13 (FOXP1) for all cancers (**a**), ERG fusion-negative (**b**) and ERG fusion-positive subset (**c**) according to ERG-IHC analysis. **d** Kaplan-Meier curves for the relationship of ZIC2 immunostaining with PSA recurrence-free survival in all cancers (**a**), in ERG fusion-negative cancers (**b**), in ERG fusion-positive cancers (**c**), in PTEN normal cancers (**d**) and in PTEN deleted cancers (**e**). Log-rank *p* values

ZIC2 was one of the candidate genes for which a suitable antibody for IHC was available. ZIC2 expression was analysed by immunohistochemistry on a tissue microarray (TMA) containing more than 12,000 prostate cancer specimens (Table 4). Results were compared with tumour phenotype, BCR, ETS-related gene (ERG) status and other recurrent genomic alterations. ZIC2 expression was detectable and considered to be strong in 23.3% of cases and was absent in the majority of the tumours (76.7%) (Fig. 4a, Table 4). Loss of ZIC2 protein expression was associated with ERG-fusion positivity (*p* < 0.0001) (Fig. 4b). Loss of ZIC2 expression was also linked to Gleason grade, advanced pathological tumour (pT) stage, lymph node metastasis and higher preoperative PSA levels in all cancers (*p* < 0.0001, each) and in the subset of ERG-fusion negative tumours (Table 4, data not shown). These associations were either weaker or absent in ERG-fusion positive cancers (data not shown). Within ERG fusion-negative cancers, ZIC2 expression was also strongly associated with 6q15 and 5q21 deletions (*p* < 0.001) (Fig. 4c). Loss of ZIC2 expression was associated with adverse outcome and correlated with significantly shorter time to biochemical recurrence in all cancers, independent of ERG and PTEN (Fig. 4d). The prognostic value of ZIC2 proved to be independent from established clinicopathological variables including Gleason, stage, nodal stage and PSA. Overall, ZIC2 was identified as an excellent marker and might provide clinically useful predictive information by identification of aggressive prostate cancer subsets.

**Table 4:** Association between ZIC2 immunostaining results and prostate cancer phenotype in tissue micro-arrayed cancers

Parameter	*n* evaluable	Negative (%)	Positive (%)	*p* value	Bonferroni correction
All cancers	12,581	76.7	23.3		
Tumour stage					
pT2	7994	74.6	25.4	< 0.0001	0.000006
pT3a	2837	78.7	21.3		0.000018
pT3b-pT4	1700	83.1	16.9		0.000029
Gleason grade					
≤ 3 + 3	2303	71.5	28.5	< 0.0001	0.000022
3 + 4	6700	75.1	24.9		0.000007
3 + 4 Tert.5	606	77.7	22.3		0.000083
4 + 3	1238	81.4	18.6		0.000040
4 + 3 Tert.5	907	85.6	14.4		0.000055
≥ 4 + 4	731	87.1	12.9		0.000068
Lymph node metastasis					
N0	7459	77.6	22.4	< 0.0001	0.000007
N+	932	86.3	13.7		0.000054
Preop. PSA level (ng/ml)					
< 4	1479	75.8	24.2	< 0.0001	0.000034
4–10	7470	75.4	24.6		0.000007
10–20	2642	79.2	20.8		0.000019
> 20	916	81.3	18.7		0.000055
Surgical margin					
Negative	10,018	75.8	24.2	< 0.0001	0.000005
Positive	2519	80.3	19.7		0.000020

## Discussion

In the present study, we have identified methylation differences related to PCa prognosis and subsequently showed that methylation-based prediction of PCa prognosis using random forest-based modelling is feasible with high accuracy.

PCa is the most prevalent cancer among men in Germany. With a 5-year survival rate of 91%, PCa is a cancer type with comparably good prognosis (German Cancer Registry). Nevertheless, biomarkers predicting the prognosis of PCa are needed for an efficient clinical management, to avoid overtreatment of cases with indolent disease and to identify patients who develop aggressive forms and require chemotherapy [34].

DNA methylation is an excellent source for biomarker development, since it is a stable modification and can be quantitatively determined in clinical samples with high throughput and precision and relatively low cost [35]. Previous studies trying to establish a methylation-based classifier for prostate cancer mostly used a preselected set of genes [36, 37] or used high Gleason score as an outcome [38, 39]. Here, we are presenting a genome-wide approach, with PSA recurrence-free survival as an endpoint. One limitation of our study is the use of the Illumina 450k platform for biomarker selection, which limits methylation analyses to preselected CpG sites on the 450k array (enriched for CpG islands and flanking regions, bioinformatically predicted enhancers, DNase I hypersensitive sites, and validated differentially methylated regions [40]). Future studies using whole genome bisulfite sequencing (WGBS) of all > 29 million CpG sites in the human genome will allow identification of additional biomarkers.

Our discovery cohort consisted of 70 patients, 35 with good and 35 with poor prognosis. After cell type adjustments, our cut-off criteria for selection of differentially methylated CpG sites were absolute methylation differences > 10% and an FDR-adjusted *p* value < 0.2. Altogether, 402 DMS and the PEPCI score for tumour aggressiveness [9] were included in the prediction model. Our random forest-based model demonstrated excellent performance with the discovery cohort (AUC 95%). We were able to validate our results using the ICGC PCa cohort of early and late prostate cancer (AUC 77.1%), with slightly worse performance using the TCGA PRAD dataset (AUC 68.7%). Different reasons might contribute to the lower performance with the TCGA PRAD cohort, such as possibly different definitions of PSA recurrence-free survival and the generally high Gleason score and high tumour stage of the TCGA patients. Other genome-wide studies have faced similar problems using TCGA as a validation set [38, 41]. Nevertheless, for both ICGC and TCGA validation cohorts, the resulting prognostic subgroups had highly significantly different survival rates.

We compared the performance of our model with commercially available, RNA expression-based genomic tests. Using RNA-seq data available for the TCGA PRAD cohort, we generated sums of *Z*-scores for the gene lists included in the Decipher, OncotypeDX and Prolaris tests, as described by Wei et al. [42]. Prolaris outperformed Decipher and OncotpyeDX with an AUC of 64.6% versus 51.5% and 50.8% for Decipher and OncotypeDX **(**Additional file 7: Figure S5). Our methylation-based classifier showed a higher AUC (68.7%) with the TCGA PRAD cohort. The low performance of Decipher and OncotypeDX might be due to the fact that the commercially available tests were not designed for RNA-seq data.

We identified significantly more loss than gain in methylation associated with PCa progression and could map the majority of our top selected candidate biomarker to PMDs. A recent small breast cancer study concluded that loss of methylation in PMDs might be more valuable as diagnostic than prognostic biomarker [43]. Generally, loss of methylation of candidate DMS located in PMDs might be more informative on larger-scale methylation changes in these late-replicating heterochromatic regions than have functional relevance on the expression of the associated genes. Accordingly, Brinkman et al. concluded that PMDs commonly did not overlap with tumour suppressor genes in breast cancer [43]. Our findings, in conjunction with WGBS data on PCa [21], support a more intensive analysis of the prognostic relevance of PMD methylation in PCa.

A recent proteomics-based biomarker study of curable prostate cancer reported a stronger link of DNA methylation status to protein than mRNA abundance [44]. In line with these findings, we performed a validation of the clinical impact of ZIC2 as one of the candidate genes on more than 12,000 micro-arrayed PCa cases. The *zinc finger of the cerebellum* (ZIC) family of genes consists of five human homologues ZIC1–5 [45]. ZIC family members inhibit TCF4/β-catenin and interact with GLI signalling [46]. ZIC2 is related to the sonic hedgehog pathway. Its oncogenic role was described in epithelial ovarian cancer [47], hepatocellular carcinoma [48] and pancreatic cancer [49]. Our IHC validation indicated a particularly strong adverse prognostic value of ZIC2 expression loss, including early biochemical recurrence and high Gleason grade. Of note, an eminent weakness of Gleason grading is the high inter-observer variability between pathologists that generally exceeds 30% [50]. In this study, the original Gleason grade from the patient’s files was used for statistical analyses. From 2005 on, in our department, Gleason grading was performed almost exactly as recommended by the WHO 2016 classification [51]. ZIC2 analysis, thus, appears to be of high value for distinguishing between patients with more or less aggressive forms of the disease and may be useful to select patients for active surveillance.

There are some limitations connected to our study. The patient cohort used for candidate gene identification was selected from patients subjected to curative radical prostatectomy and did not include individuals with advanced castration-resistant cancers who have the worst prognosis. Also, a 5-year recurrence-free interval as defined for our good prognosis group might be too short to select only patients with the best possible prognosis. Thus, it cannot be excluded that some relevant candidate genes may have been missed by our approach. The same might also apply to the 17,000 cancer validation set, which is also limited to prostatectomy specimens. An optimal validation set would have been made up from biopsy specimens which precisely represent the kind of samples that are available for molecular analysis when a therapy decision has to be taken. However, needle biopsies are precious material that is exhausted after only a few analyses. The 0.6-mm TMA punches used in our study very much resemble the size of needle biopsies. This makes them probably well-suited to reflect the diagnostic problems connected to needle biopsy analysis including a possible selection bias, heterogeneity issues and the limited amount of cancer cells available for analysis.

## Conclusions

We present a candidate selection of cancer progression-related CpG methylation changes, as well as a classification model to predict aggressive behaviour of PCa. This model, with further tuning, might help in decision making related to the treatment of prostate cancer patients. The effect of candidate CpG site methylation on gene expression helps to pinpoint further genes, which play an important role in prostate cancer development. Ranking of the selected CpG sites and associated genes allowed selection of candidate biomarkers for validation by IHC. We identified loss of ZIC2 expression as a promising prognostic biomarker for PCa.

## Methods

### Study population

In order to build a classifier that predicts the patients’ outcome the best, a highly selected group of patients was included in the study. Sample selection was based on the following criteria: good prognosis indicated by the presence of organ-confined disease (pT2) and lack of biochemical prostate-specific antigen (PSA)-based recurrence (BCR) for at least 5 years. In contrast, poor prognosis is defined as systemic presence of metastatic disease, indicated by BCR within 3 years and no response to local radiation therapy. Initially, 84 patients were selected.

A pathologist selected FFPE tissue blocks containing tumour-rich areas (≥ 70% tumour cells) for analysis. Three tissue punches (0.6 mm × 3 mm) were taken of each tissue block, and genomic DNA was isolated using the AllPrep® DNA/RNA FFPE kit (Qiagen). DNA was submitted to the DKFZ Genome and Proteome core facility for Illumina 450k Methylation analyses. After removing samples and DNA methylation profiles with low quality, the study included 35 patients with good and 35 patients with bad prognosis (Additional file 1: Table S1).

### Validation datasets

The ICGC PCa cohort has been described earlier [9]. Clinical information for the TCGA PRAD cohort was downloaded from cBioPortal in June 2018 (Table 2). For the subcohorts, patients were selected as good prognosis patients by lack of BCR within 5 years and a disease stage pT2 and as poor prognosis patients when suffering from BCR within 3 years and having a stage pT3 or pT4. Consensus androgen receptor (AR) binding sites (*n* = 8162) were defined by Stelloo et al. [17] based on AR ChIP-seq data for 100 prostate carcinomas. Genomic distances of DMS-associated gene TSSs were calculated using the middle point of the nearest region. Prostate cancer WGBS data was accessed at GEO accession number GSE104789 [21]. Information on common PMDs was derived from [22].

### DNA methylation processing

DNA methylation was assessed using the Illumina HumanMethylation450 Array. The methylation data was processed using the RnBeads R package [52]. Probes with SNPs (dbSNP 144) overlapping with the C nucleotide of the CG site and having MAF> 0.01 (28,722 probes) were excluded. Probes with high likelihood of false hybridization (28,736 probes, as defined in RnBeads) were also removed. Quality filtering was performed using the Greedycut algorithm, which removed 21,040 probes and 11 samples. Additional 969 non-CpG probes and 9229 probes located on the sex chromosomes were removed. No normalization or background correction was used.

During the analysis, a batch effect was observed between data from fresh frozen (TCGA PRAD and ICGC PCa cohort) and formalin-fixed tissue (discovery cohort). In order to have a generalizable model, we avoided shifting the beta values as would happen with batch correction methods. Instead, we used principal component analysis (PCA) on the top 10,000 most variable CpG sites to identify the probes affected by this effect. This was done using two independent datasets containing formalin-fixed [53] or fresh frozen tissue [8] and removed the top 5000 sites captured by PC2, the main principal component affected by the sample type. The PEPCI score and the basal, stromal, luminal, T-luminal and immune cell composition were estimated using the PEPCI R package [9]. Linear models of the limma package [54] were applied to identify differentially methylated probes after adjustments for age, basal, stromal and immune cell content. CpG sites with FDR-adjusted *p* values < 0.2 and mean methylation difference > 0.1 (10%) were used to build the model. Enrichment analysis of the significantly methylated sites, promoters and genes were performed with EpiAnnotator [55]. Annotation of the most important CpG sites of the random forest model was done using the GREAT tool [19].

### Random forest classifier

A random forest-based classifier was built using the randomForest R package, which is based on the algorithm of Breiman and Cutler [16]. Random forest is a learning method that constructs numerous decision trees and outputs the classes (in case of classification) of the individual trees. The predicted class of the input instance will be decided upon majority vote (schematic principle in Additional file 8**:** Figure S6). Each tree was built on a bootstrap training set, which represents about two thirds of the discovery cohort with replacement. Out-of-bag (OOB) error was used to measure the performance of the model on the training set. Classification of the instances left out (OOB samples) was used to estimate a generalization error (OOB error). The OOB error will give an unbiased estimate of the current classification error, while the bagging method will decrease the chance of overfitting.

Two variable importance scores are used in random forest. The mean decrease in accuracy reflects a variable importance measure to assess the prediction strength of each predictor variable. When a tree is grown, the OOB samples are used to calculate the error rate. Then, the values of a given predictor variable are randomly permuted and the error rate is calculated again. The decrease in accuracy caused by the permutation is averaged over all trees. The mean decrease in Gini score gives the improvement in the split-criterion at each split in each tree [20].

Twenty different models were trained as follows: data was randomly split into training (80%) and test (20%) set. The model was trained on the training set, with 10,000 trees and 19 variables to select randomly for each tree. Prediction accuracy was measured on the test set. The results were collected and the best performing model was selected. This model was then optimized for the number of variables selected for each tree. For variable selection, CpG sites were ranked based on mean decrease in accuracy and mean decrease in Gini scores [20].

Validation of the classifier was performed using the TCGA PRAD and the ICGC cohort of early and late prostate cancer. TCGA-PRAD DNA methylation data was downloaded from the GDC portal (https://portal.gdc.cancer.gov/) legacy archive in .idat format. The performance was evaluated with ROC curve analysis, using the ROCR R package [56] and Kaplan-Meier curves for the validation datasets and individual candidate CpG sites.

### Candidate selection

Based on our model, the top-rated candidates underwent a further selection to identify the ones with the highest possibility to perform well as a protein expression-based biomarker. First, we used the GREAT tool and the gene annotation of the Illumina 450k methylation array to identify gene-CpG site associations, by selecting the genes closest to the sites. The CpG sites in close vicinity to transcription start sites (± 2 kb) were preferred, to enhance the potential functional relevance for correlated gene/protein expression changes. Finally, the selection was based on the mean decrease in the Gini score (cutoff > 0.1).

### Genomic risk scores

Risk scores for TCGA-PRAD based on the gene expression panels of Decipher, Oncotype DX and Prolaris tests were calculated as described in [42]. TCGA-PRAD RNA-Seq HTSeq counts were downloaded from GDC portal (https://portal.gdc.cancer.gov/). Gene-based *Z*-scores were calculated for the 19, 12 and 31 genes of the respective panels. The sum of the scores was used as risk scores.

### Validation of candidate genes by immunohistochemistry (IHC)

#### Patients

Radical prostatectomy specimens were available from 17,747 patients undergoing surgery between 1992 and 2017 at the Department of Urology and the Martini Clinics at the University Medical Centre Hamburg-Eppendorf (Additional file 9: Table S3). All prostate specimens were analysed according to a standard procedure, including complete embedding of the entire prostate for histological analysis [57]. Histo-pathological data was retrieved from the patient files, including tumour stage, Gleason grade, nodal stage and resection margin status. Gleason grading was performed already from 2005 on as outlined later in the 2016 WHO recommendations with minor modifications, i.e., we have a conservative position to define irregular glands as Gleason 4. Follow-up data were available for a total of 14,464 patients with a median follow-up of 48 months (range 1 to 241 months; Additional file 9: Table S3). PSA values were measured in regular intervals following surgery, and PSA recurrence was defined as the measurement of a postoperative PSA of ≥ 0.2 ng/ml and increasing. The TMA manufacturing process was described in detail earlier [58]. In short, one 0.6-mm core was taken from a tumour-containing tissue block from each patient. The molecular database attached to this TMA contained results on ERG expression in 10,711 [3], *ERG* break apart FISH analysis in 7122 (expanded from [59]), deletion status of 5q21 (*CHD1*) in 7932 (expanded from [60]), 6q15 (*MAP 3 K7*) in 6069 (expanded from [61]), 10q23 (*PTEN*) in 6704 (expanded from [62]) and 3p13 (*FOXP1*) in 7081 (expanded from [63]) cancers.

#### Immunohistochemistry

Freshly cut TMA sections were immunostained on one day and in one experiment. Slides were deparaffinised and exposed to heat-induced antigen retrieval for 5 min in an autoclave at 121 °C in pH 7.8 Tris-EDTA-citrate buffer. The primary antibody specific for ZIC2 (antibodies online, ABIN2776475) was applied at 37 °C for 60 min. Bound antibody was then visualized using the EnVision Kit (Dako, Glostrup, Denmark) according to the manufacturer’s directions. ZIC2 staining intensity was assessed as negative or positive.

#### Statistics

Statistical calculations were performed with JPM 12 software (SAS Institute Inc., NC, USA). Contingency tables and the *χ*^2^ test were performed to search for associations between molecular parameters and tumour phenotype. Survival curves were calculated according to Kaplan-Meier. The log-rank test was applied to detect significant survival differences between groups. Cox proportional hazards regression analysis was performed to test the statistical independence and significance between pathological, molecular and clinical variables. Separate multivariate analyses were performed using different sets of parameters available either before or after prostatectomy.

## Supplementary information files

**Additional file 1: Table S1.** Clinical information and cell type composition of discovery cohort samples.

**Additional file 2: Table S2.** Full list of CpG sites contributing to the model.

**Additional file 3: Figure S1.** Individual Kaplan-Meier curves. Predictive power for PSA recurrence–free survival of the full classifier, PEPCI, and individual candidate CpG sites associated with the top20 selected genes in the discovery cohort (*n* = 70). *p* values from log-rank test. Red: high methylation (above median), blue: low methylation (below median).

**Additional file 4: Figure S2.** Performance of the random forest model. The plot shows the performance of the random forest model as a function of the trees built in the model, using the generalized OOB (black) and classification error for the good (red) and poor (green) prognosis groups.

**Additional file 5: Figure S3.** Heatmap of the selected CpG sites in the ICGC prostate cancer (left) and TCGA PRAD (right) validation datasets. Each column represents a sample with predicted good or poor prognosis, while rows represent selected differentially methylated CpG sites. Annotations on the left side indicate top ranked candidate genes associated with most informative CpG sites. Low and high methylation beta values in a range from 0 to 1 are shown in a blue to red color scale. BCR: PSA-based biochemical recurrence.

**Additional file 6: Figure S4.** Localization of DMS in PMDs identified in prostate cancer by WGBS. WGBS data for three prostate cancer cases with matching benign tissue was derived from GSE104789 and uploaded to the UCSC genome browser. For comparison, common PMDs identified in eight common cancer types excluding prostate cancer [22] were displays in a color gradient from light grey to black.

**Additional file 7: Figure S5.** Specificity and sensitivity of gene expression-based prognostic tests to prognosticate PSA-based BCR for the TCGA PRAD cohort. Sums of Z-scores of RNA-seq-derived gene expression per patient were used for calculations of risk scores, as described in Ref. [42].

**Additional file 8: Figure S6.** Schematic representation of the random forest model.

**Additional file 9: Table S3.** Pathological and clinical data of the arrayed prostate cancers.

## Data Availability

Methylation data for the discovery cohort has been uploaded to GEO under accession No. GSE127985.

## Abbreviations

AUC: Area under the receiver operating characteristic (ROC) curve
BCR: Biochemical recurrence
DMS: Differentially methylated CpG site
FFPE: Formalin-fixed paraffin-embedded
IHC: Immunohistochemistry
OOB: Out-of-bag
PCa: Prostate cancer
PMDs: Partially methylated domain
PSA: Prostate-specific antigen
ROC: Receiver operating characteristic
TMA: Tissue micro-array
TSS: Transcription start site
ZIC2: Zic family member 2
