# Supplementary material for: Random forest-based modelling to detect biomarkers for prostate cancer progression
Source: Clin Epigenetics. 2019 Oct 22;11:148. doi: 10.1186/s13148-019-0736-8 (PMC6805338; doi:10.1186/s13148-019-0736-8)
Supplement: Supplementary file 3 — Additional file 3: Figure S1. Individual Kaplan-Meier curves. Predictive power for PSA recurrence–free survival of the full classifier, PEPCI, and individual candidate CpG sites associated with the top20 selected genes in the discovery cohort (n = 70). p values from log-rank test. Red: high methylation (above median), blue: low methylation (below median). [file 13148_2019_736_MOESM3_ESM.pdf]

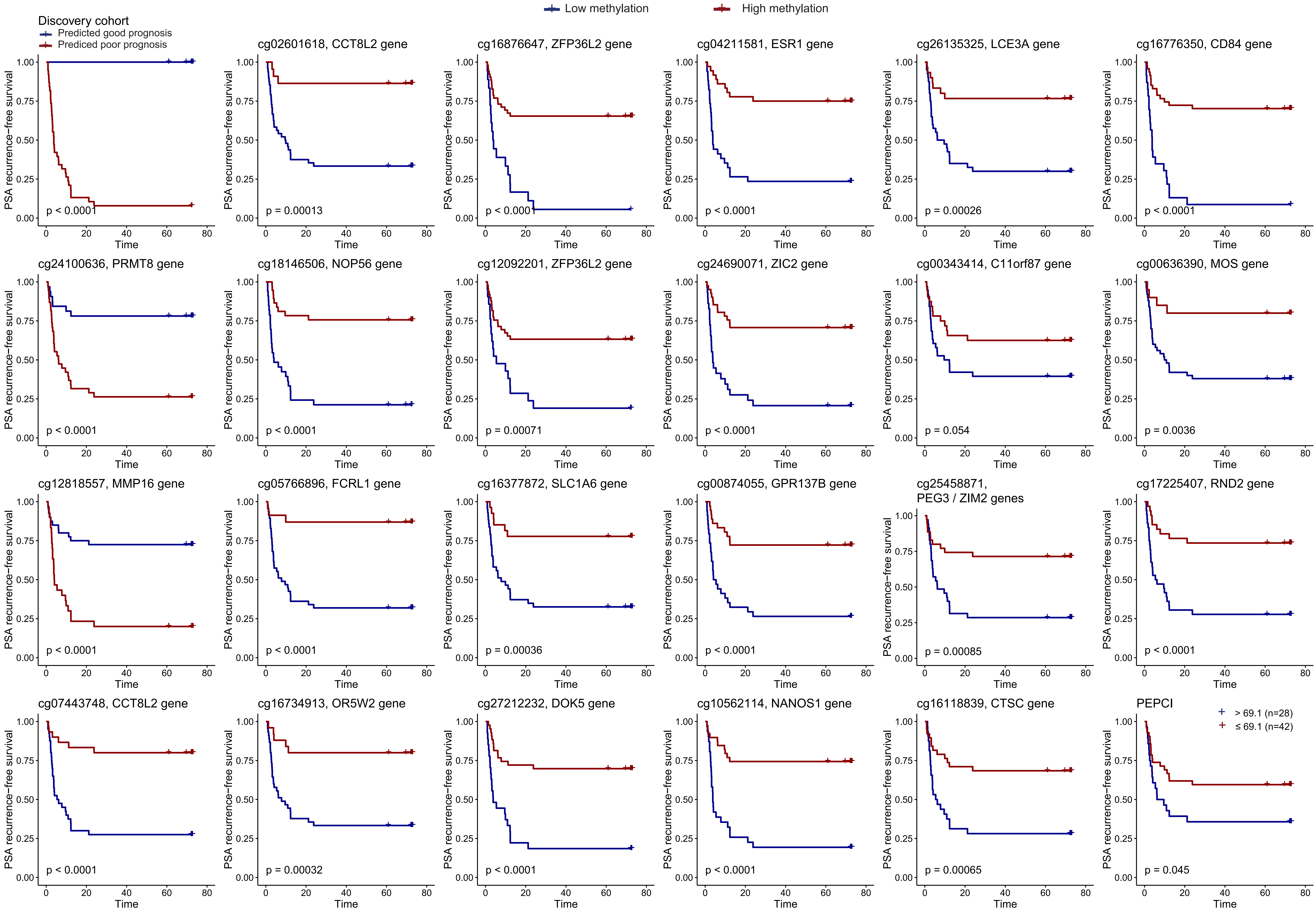

Figure S1: Individual Kaplan-Meier curves. Predictive power for PSA recurrence–free survival of the full classifier, PEPCI, and individual candidate CpG sites associated with the top20 selected genes in the discovery cohort (n=70). p values from log-rank test. Red: high methylation (above median), blue: low methylation (below median).
