# Supplementary material for: Random forest-based modelling to detect biomarkers for prostate cancer progression
Source: Clin Epigenetics. 2019 Oct 22;11:148. doi: 10.1186/s13148-019-0736-8 (PMC6805338; doi:10.1186/s13148-019-0736-8)
Supplement: Supplementary file 4 — Additional file 4: Figure S2. Performance of the random forest model. The plot shows the performance of the random forest model as a function of the trees built in the model, using the generalized OOB (black) and classification error for the good (red) and poor (green) prognosis groups. [file 13148_2019_736_MOESM4_ESM.pdf]

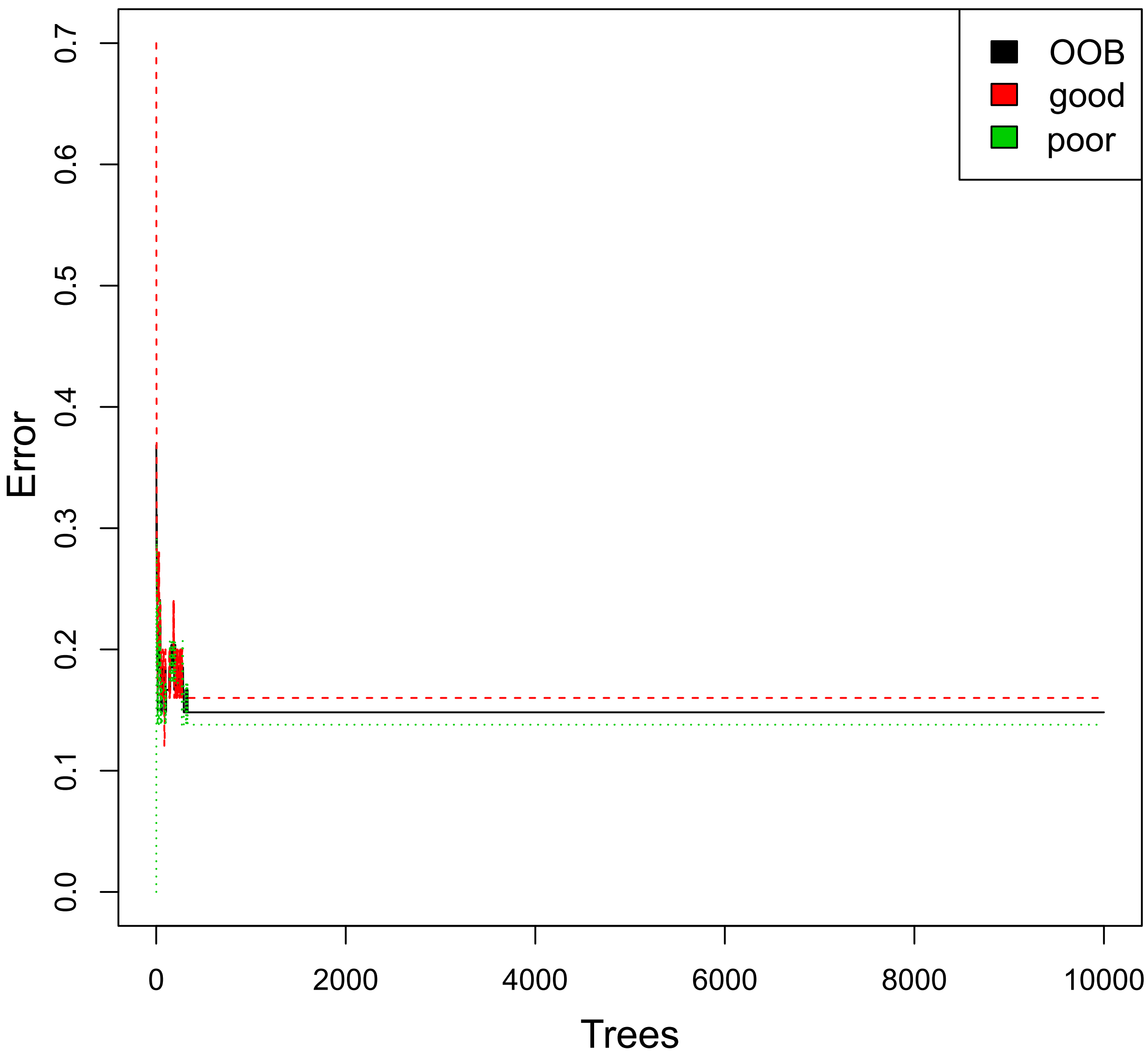

Figure S2: Performance of the random forest model. The plot shows the performance of the random forest model as a function of the trees built in the model, using the generalized OOB (black) and classification error for the good (red) and poor (green) prognosis groups.
