# Supplementary material for: Random forest-based modelling to detect biomarkers for prostate cancer progression
Source: Clin Epigenetics. 2019 Oct 22;11:148. doi: 10.1186/s13148-019-0736-8 (PMC6805338; doi:10.1186/s13148-019-0736-8)
Supplement: Supplementary file 5 — Additional file 5: Figure S3. Heatmap of the selected CpG sites in the ICGC prostate cancer (left) and TCGA PRAD (right) validation datasets. Each column represents a sample with predicted good or poor prognosis, while rows represent selected differentially methylated CpG sites. Annotations on the left side indicate top ranked candidate genes associated with most informative CpG sites. Low and high methylation beta values in a range from 0 to 1 are shown in a blue to red color scale. BCR: PSA-based biochemical recurrence. [file 13148_2019_736_MOESM5_ESM.pdf]

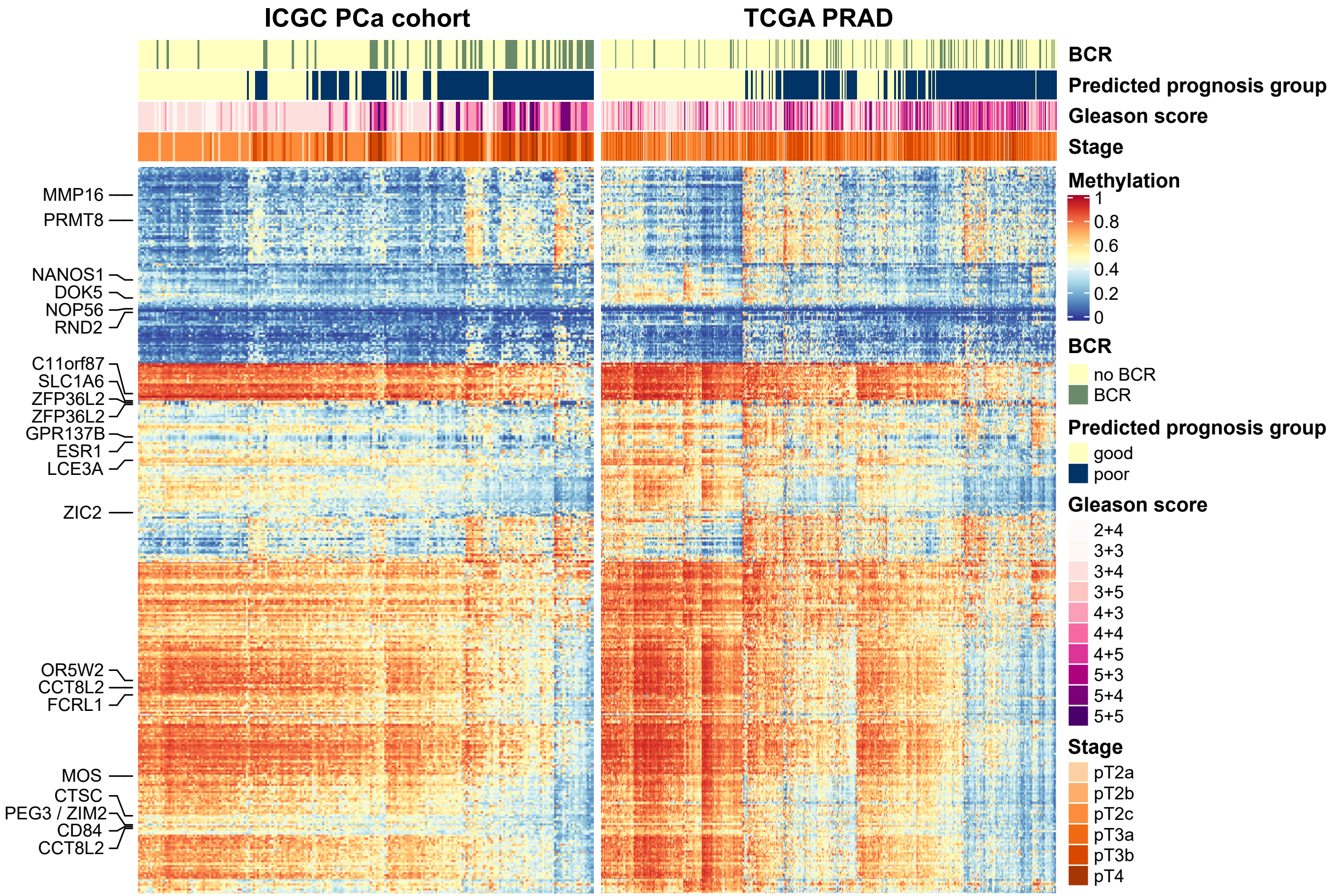

Figure S3: Heatmap of the selected CpG sites in the ICGC prostate cancer (left) and TCGA PRAD (right) validation datasets. Each column represents a sample with predicted good or poor prognosis, while rows represent selected differentially methylated CpG sites. Annotations on the left side indicate top ranked candidate genes associated with most informative CpG sites. Low and high methylation beta values in a range from 0 to 1 are shown in a blue to red color scale. BCR: PSA-based biochemical recurrence.
