# Supplementary material for: Random forest-based modelling to detect biomarkers for prostate cancer progression
Source: Clin Epigenetics. 2019 Oct 22;11:148. doi: 10.1186/s13148-019-0736-8 (PMC6805338; doi:10.1186/s13148-019-0736-8)
Supplement: Supplementary file 6 — Additional file 6: Figure S4. Localization of DMS in PMDs identified in prostate cancer by WGBS. WGBS data for three prostate cancer cases with matching benign tissue was derived from GSE104789 and uploaded to the UCSC genome browser. For comparison, common PMDs identified in eight common cancer types excluding prostate cancer [22] were displays in a color gradient from light grey to black. [file 13148_2019_736_MOESM6_ESM.pdf]

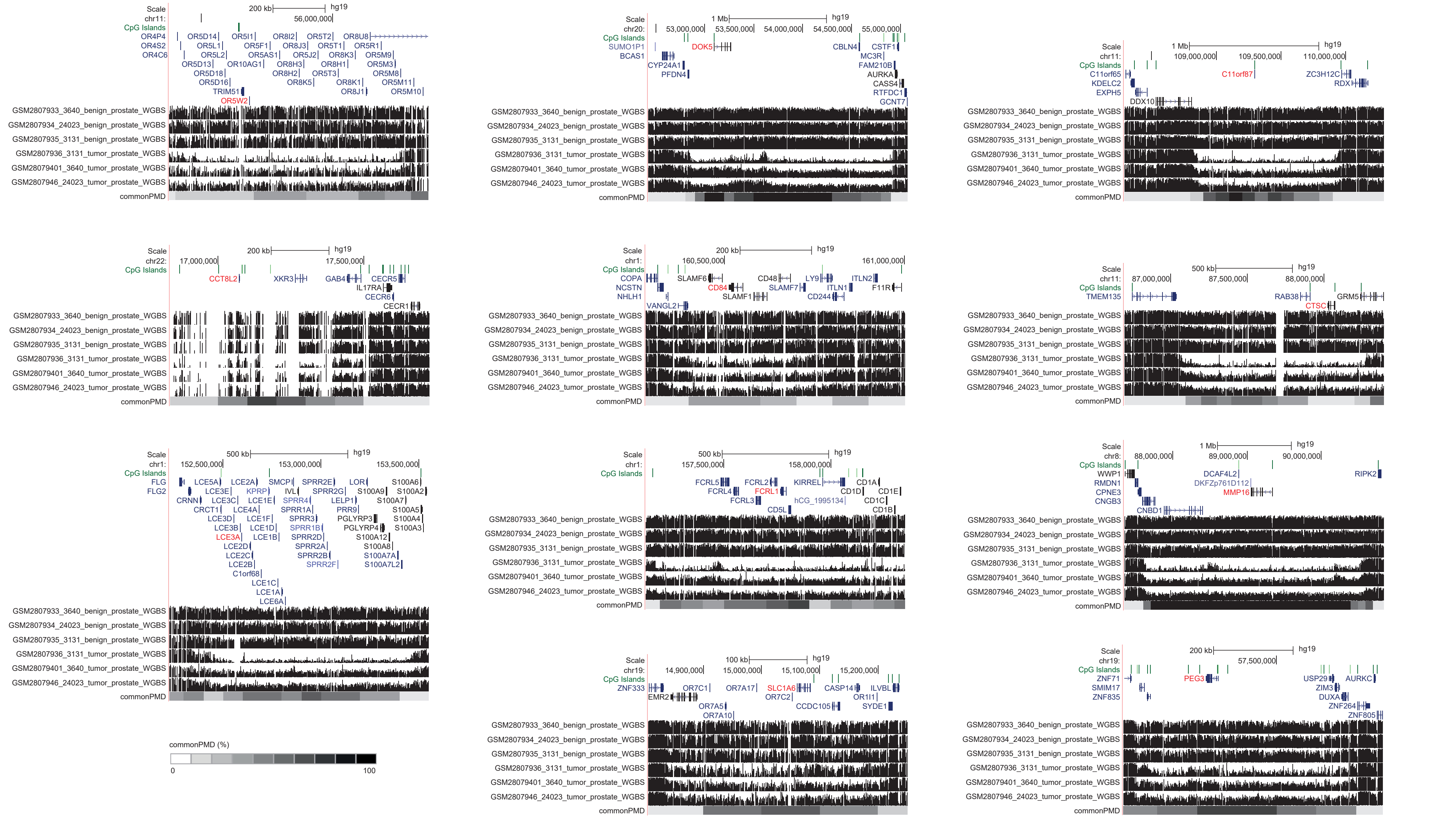

Figure S4: Localization of DMS in PMDs identified in prostate cancer by WGBS. WGBS data for three prostate cancer cases with matching benign tissue was derived from GSE104789 and uploaded to the UCSC genome browser. For comparison, common PMDs identified in eight common cancer types excluding prostate cancer [22] were displays in a color gradient from light grey to black.
