# Supplementary material for: Random forest-based modelling to detect biomarkers for prostate cancer progression
Source: Clin Epigenetics. 2019 Oct 22;11:148. doi: 10.1186/s13148-019-0736-8 (PMC6805338; doi:10.1186/s13148-019-0736-8)
Supplement: Supplementary file 7 — Additional file 7: Figure S5. Specificity and sensitivity of gene expression-based prognostic tests to prognosticate PSA-based BCR for the TCGA PRAD cohort. Sums of Z-scores of RNA-seq-derived gene expression per patient were used for calculations of risk scores, as described in Ref. [42]. [file 13148_2019_736_MOESM7_ESM.pdf]

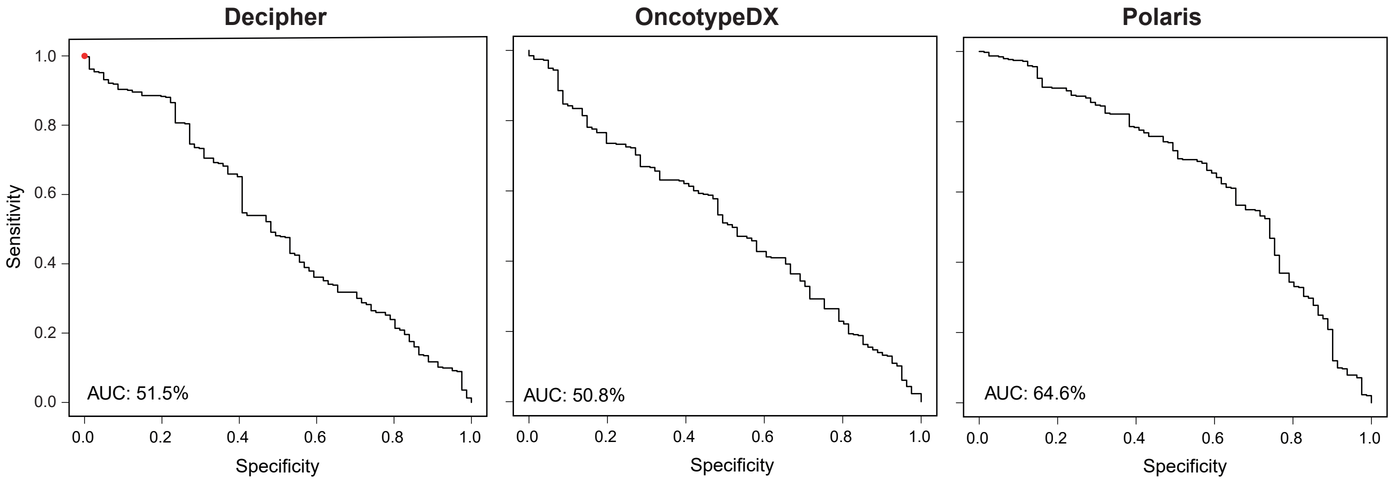

Figure S5: Specificity and sensitivity of gene expression-based prognostic tests to prognosticate PSA-based BCR for the TCGA PRAD cohort. Sums of Z-scores of RNA-seq-derived gene expression per patient was used for calculations of risk scores, as described in Ref. [42].
