# Supplementary figures and images for: Random forest-based modelling to detect biomarkers for prostate cancer progression
Source: Clin Epigenetics. 2019 Oct 22;11:148. doi: 10.1186/s13148-019-0736-8 (PMC6805338; doi:10.1186/s13148-019-0736-8)

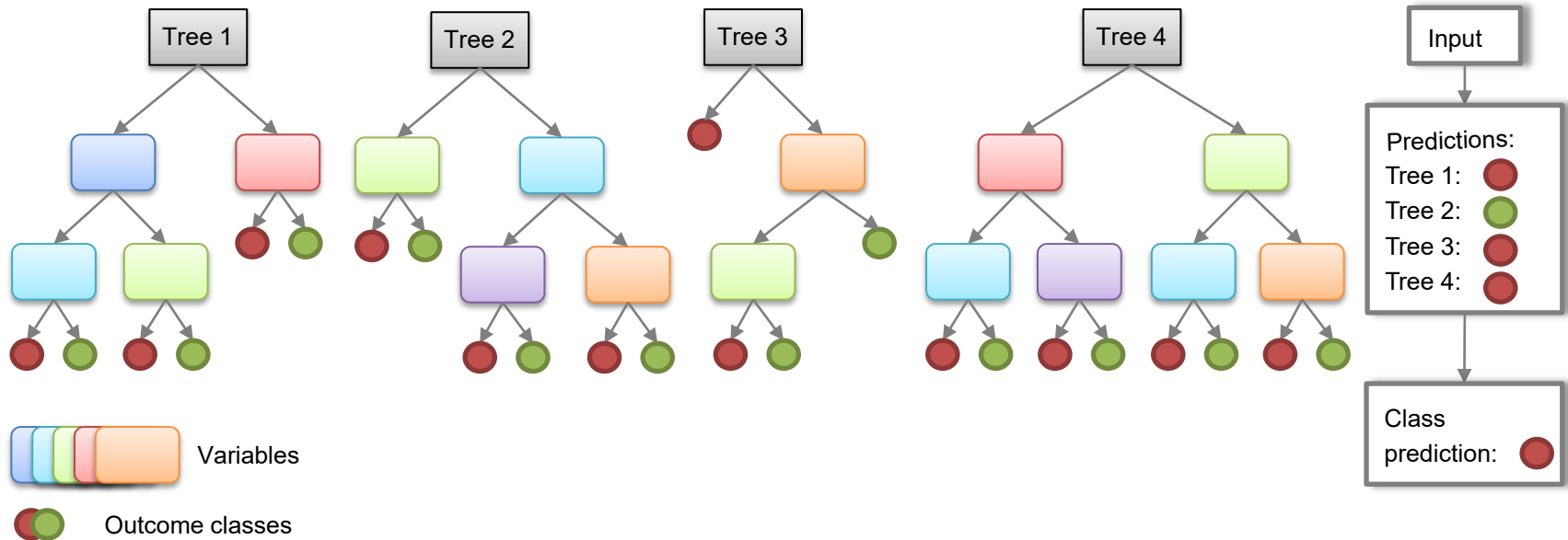

**Figure S5:** Schematic representation of the random forest model

Supplement: Supplementary file 8 — Additional file 8: Figure S6. Schematic representation of the random forest model. [file 13148_2019_736_MOESM8_ESM.pdf]
