# Supplementary material for: Random forest-based modelling to detect biomarkers for prostate cancer progression
Source: Clin Epigenetics. 2019 Oct 22;11:148. doi: 10.1186/s13148-019-0736-8 (PMC6805338; doi:10.1186/s13148-019-0736-8)
Supplement: Supplementary file 9 — Additional file 9: Table S3. Pathological and clinical data of the arrayed prostate cancers. [file 13148_2019_736_MOESM9_ESM.pdf]

Table S3: Pathological and clinical data of the arrayed prostate cancers

Percent in the column "Study cohort on TMA" refers to the fraction of samples across each category. Percent in column "Biochemical relapse among categories" refers to the fraction of samples with biochemical relapse within each parameter in the different categories.

	No. of patients (%)	
	Study cohort on TMA (n=17747)	Biochemical relapse among categories
**Follow-up (months)**		
n	14464 (81.5%)	3612 (25%)
Mean	56.3	-
Median	48	-
**Age (years)**		
≤50	433 (2.4%)	66 (15.2%)
51-59	4341 (24.5%)	839 (19.3%)
60-69	9977 (56.4%)	2073 (20.8%)
≥70	2936 (16.6%)	634 (21.6%)
**Pretreatment PSA (ng/ml)**		
<4	2225 (12.6%)	313 (14.1%)
≥4-10	10520 (59.6%)	1696 (16.1%)
≥10-20	3662 (20.8%)	1043 (28.5%)
>20	1231 (7%)	545 (44.3%)
**pT stage (AJCC 2002)**		
pT2	11518 (65.2%)	1212 (10.5%)
pT3a	3842 (21.7%)	1121 (29.2%)
pT3b	2233 (12.6%)	1213 (54.3%)
pT4	85 (0.5%)	63 (74.1%)
**Gleason grade**		
≤3+3	3570 (18.1%)	264 (7.4%)
3+4	9336 (47.4%)	1436 (15.4%)
3+4 Tert.5	1697 (8.6%)	165 (9.7%)
4+3	2903 (14.7%)	683 (23.5%)
4+3 Tert.5	1187 (6%)	487 (41%)
≥4+4	999 (5.1%)	531 (53.2%)
**pN stage**		
pN0	10636 (89.4%)	2243 (21.1%)
pN+	1255 (10.6%)	700 (55.8%)
**Surgical margin**		
Negative	14297 (80.8%)	2307 (16.1%)
Positive	3388 (19.2%)	1304 (38.5%)

NOTE: Numbers do not always add up to 17747 in the different categories because of cases with missing data. Abbreviation: AJCC, American Joint Committee on Cancer.
